# Supplementary material for: A Y-chromosome shredding gene drive for controlling pest vertebrate populations
Source: eLife. 2019 Feb 15;8:e41873. doi: 10.7554/eLife.41873 (PMC6398975; doi:10.7554/eLife.41873)
Supplement: Supplementary file 1. [file elife-41873-supp1.docx]

**Supplementary File 1.** Details of the gRNA sequences (a) and qPCR primers (b) used for the empirical study of Y-shredding efficiency.

**(a) gRNA sequences used**

| Name | gRNA sequences 5’-3’ | On target site positions in Y |
| --- | --- | --- |
| Centro 37X-A | TATTAACTCCTGTATATAAGATG | 4.065.156 to 4.159.149 |
| Centro 37X-B | CATGCCATGTTCTACAACGTTCA | 4.075.040 to 4.150.309 |
| Centro 59X | TTGTTACTCATTCTTCGACAATG | 4.083.362 to 4.149.443 |
| Long arm 225X | ACCACTTCGACCTATGGGAGTGA | 4.330.370 to 90.390.289 |
| Long arm 243X | CAGAATACACTTGGCAGGGACCT | 4.596.416 to 89.920.350 |
| Long arm 256X | ATTTGCCAGGCAATGCTGAACTA | 4.663.227 to 90.542.639 |

**(b) qPCR primers**

| qPCR | F (5’-3’) | R (5’-3’) | Position |
| --- | --- | --- | --- |
| *Uba1y* (Y short arm) | GGCCACAGACTTGGGCCGAC | TGCCTTGTGGTGCCTGTGGC | chrY: 831.667 - 831.891 & 681.963 - 682.187 |
| *Erdr1* (Y long arm) | CTGACTGCGTACAGAAATGTCC | GGAAGACACACACACATCTGCA | chrY: 90.822.353 - 90.822.426 & 90.816.416 - 90.816.489 |
| *Sox1* | GACTTGCAGGCTATGTACAACATC | CCTCTCAGACGGTGGAGTTATATT | Chr8 |
